# High mobility group protein 2 (HMGA2) is highly expressed in a broad range of benign and malignant tumors

**DOI:** 10.1007/s00428-025-04142-1

**Published:** 2025-06-16

**Authors:** Viktoria Chirico, Fatih Baybars Ergüven, Katharina Möller, Florian Lutz, Florian Viehweger, Martina Kluth, Claudia Hube-Magg, Christian Bernreuther, Guido Sauter, Andreas H. Marx, Ronald Simon, Frank Jacobsen, Patrick Lebok, Till S. Clauditz, Waldemar Wilczak, Morton Freytag, Viktor Reiswich, David Dum, Andrea Hinsch, Simon Kind, Andreas M. Luebke, Ria Schlichter, Sören Weidemann, Till Krech, Stefan Steurer, Christoph Fraune, Sarah Minner, Natalia Gorbokon, Maximilian Lennartz, Eike Burandt, Anne Menz

**Affiliations:** 1 Institute of Pathology, University Medical Center Hamburg‐Eppendorf, Martinistrasse 52, 20246 Hamburg, Germany; 2 Department of Pathology, Academic Hospital Fuerth, 90766 Fuerth, Germany; 3 Institute of Pathology, Clinical Center Osnabrueck, Osnabrueck, Germany

**Keywords:** HMGA2, Immunohistochemistry, Neoplastic tissue, Drug target, Tissue microarray

## Abstract

High mobility group protein 2 (HMGA2) is an essential component of the enhanceosome that regulates gene transcription during organ development, and its re-expression in adult tissues is often linked to tumor formation and progression. To investigate HMGA2’s role in cancer, a tissue microarray of 18,582 samples from 154 tumor types and 608 samples from 76 normal tissues was analyzed. HMGA2 expression was generally higher in cancer than in normal tissues. Of the 15,915 tumor samples, 37.5% showed HMGA2 positivity: 12% weak, 11.5% moderate, and 13.9% strong. HMGA2 was detected in at least one case in 118 of 144 tumor categories, with strong staining in 92 categories. The frequency of HMGA2 positivity was highest in cancers of the ovary and the endometrium (52.6–92.6%), thyroidal neoplasms (53.4–95%), salivary gland neoplasms (66.7–98%), and non-seminomatous testicular germ cell tumors (73.5–93.5%). High-level HMGA2 staining was associated with invasive tumor growth of urinary bladder cancers (*p* < 0.0001); high-grade, advanced pT, metastasis, and poor overall survival (*p* < 0.0001 each) in clear cell renal cell carcinoma (RCC); nodal metastasis in papillary RCC (*p* = 0.0099); as well as nodal metastasis in papillary thyroid cancer (*p* = 0.0063). Low HMGA2 expression was linked to microsatellite instability in colorectal cancer (*p* = 0.0002) and HPV infection in squamous cell carcinomas (*p* < 0.0001). It is concluded that HMGA2 is highly expressed in a very broad range of tumor entities. These findings emphasize a potential role of HMGA2 as a drug target and suggest utility for HMGA2 IHC for the distinction of neoplastic from non-neoplastic tissues in several organs.

**Supplementary Information:**

The online version contains supplementary material available at 10.1007/s00428-025-04142-1.

## Introduction

The high mobility group protein 2 (HMGA2) is a small protein of less than 12 kDa, which can bind to AT-rich binding sites in the DNA minor groove [1]. These bindings affect DNA conformation and modify the transcription of numerous genes [2]. HMGA2 appears to be essential for cell growth regulation. HMGA2 knock-out in mice results in diet-induced obesity while certain HMGA2 mutations lead to unusually small-sized mice [3]. Genome-wide association studies found a relationship between the height of humans and HMGA2-linked SNPs [4–9]. HMGA2 is preferentially expressed during organogenesis while its expression is reduced in adult tissues [1, 10]. However, HMGA2 is often re-expressed in tumors, where it can promote cancer cell proliferation [11], cell motility [12], epithelial-mesenchymal transition [13–16], acquisition of cancer stem cell properties [17], and resistance to chemotherapeutic agents [18]. It is coded by the HMGA2 gene at 12q13-15, located about 2.8 MB proximal to MDM2 [1]. Causes for HMGA2 re-expression in neoplastic tissues, for example, include 12q13-15 amplification [19], gene fusions [19–22], promoter hypomethylation [23], or loss of miRNA regulation [24].

**Fig. 1:**
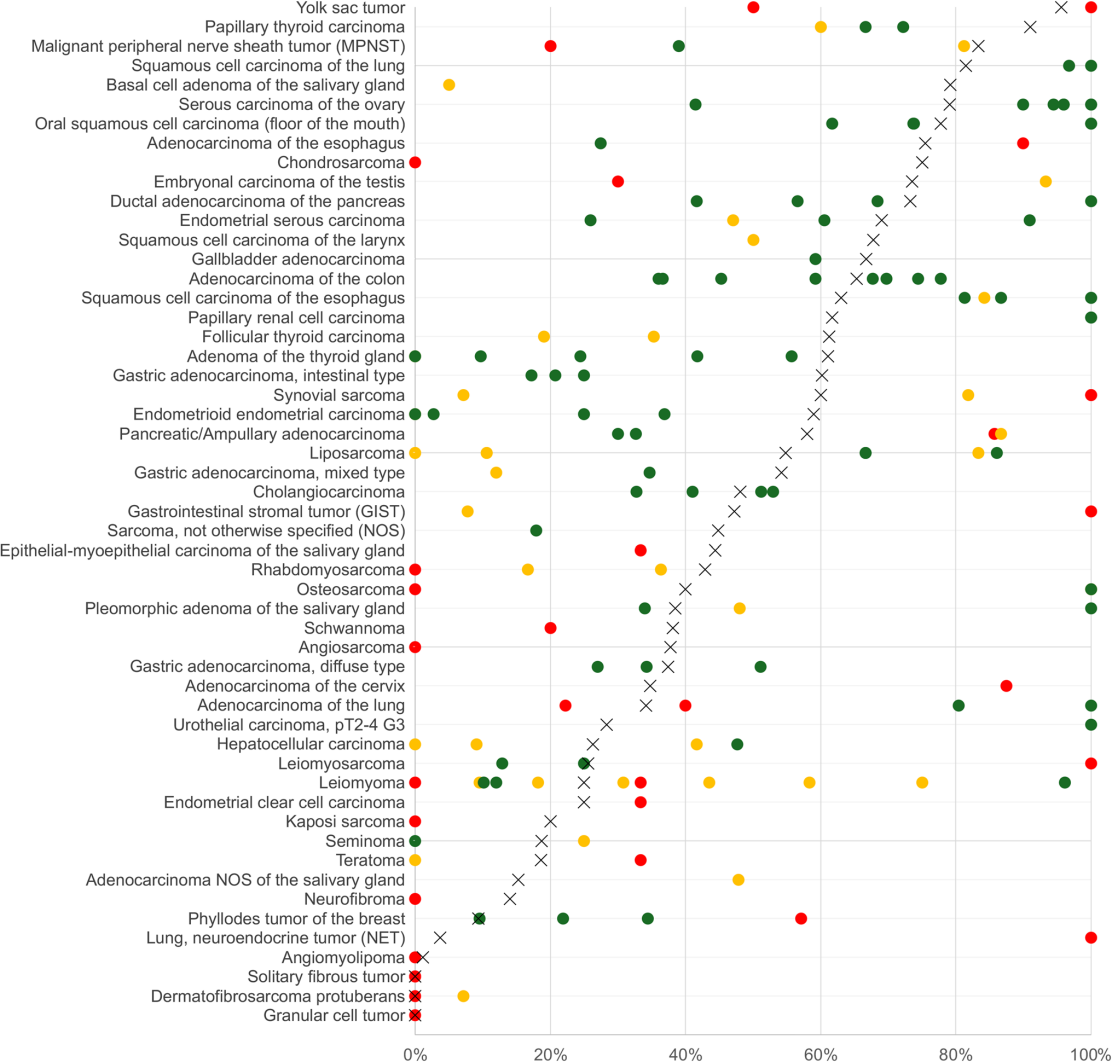
Comparison of own findings with HMGA2 data from previous literature. An “X” indicates the fraction (%) of HMGA2 positive cancers in the present study, dots indicate the reported frequencies from the literature for comparison: red dots mark studies with ≤ 10 analyzed tumors, yellow dots mark studies with 11 to 25 analyzed tumors, and green dots mark studies with > 25 analyzed tumors

Studies using immunohistochemistry (IHC) to evaluate HMGA2 in cancer have suggested considerable clinical utility of HMGA2 analysis. Associations between high levels of HMGA2 and poor prognosis and/or unfavorable tumor phenotype were found in various tumor entities [14, 25–40]. Because frequent high-level expression of HMGA2 was observed in individual tumor types, some authors have demonstrated HMGA2 positivity by IHC as a diagnostic feature for the diagnosis of pleomorphic adenoma and carcinoma ex pleomorphic adenoma of salivary glands [41], aggressive vulvovaginal angiomyxoma [42, 43], testicular yolk sac tumor [44], lipoma [45], uterine carcinoma [46], esophageal squamous cell carcinoma [47], follicular thyroid carcinoma [48], and dedifferentiated liposarcoma [49]. However, the diagnostic role of HMGA2 IHC cannot be fully assessed yet as many tumor entities have so far not been examined for HMGA2 expression and data are controversial for most tumor entities that have been analyzed in multiple studies (Fig. 1). For example, reported HMGA2 positivity rates range from 14.3 to 81.3% of malignant peripheral nerve sheath tumors (MPNST) [49, 50], 0 to 83.3% of liposarcoma [49, 51], 0 to 100% of osteosarcoma [26, 49], 0 to 96.2% of leiomyoma [49, 52], 7.14 to 100% of synovial sarcoma [49, 53], 12.9 to 100% of leiomyosarcoma [49, 54], 30 to 93.3% of embryonal carcinoma of the testis [55, 56], 22.2 to 100% of pulmonary adenocarcinomas [57, 58], 27.5 to 90% of esophageal adenocarcinomas [29, 47], 0 to 36.8% of endometrioid endometrium carcinomas [46, 59], 41.7 to 100% of ductal pancreatic adenocarcinoma [14, 25], and 36 to 77.8% of colorectal adenocarcinomas [34, 60]. Technical factors, such as staining protocols and antibodies used, different thresholds to determine positivity, as well as a possible selection bias with respect to the analyzed tumors may have caused these discrepancies.

To better understand the potential diagnostic utility and the potential prognostic role of HMGA2 expression analysis in different cancer types, more than 18,000 tissue samples from 154 different tumor types and subtypes, and 76 non-neoplastic tissues, were evaluated by IHC in a tissue microarray (TMA) format.

## Materials and methods

### Tissue microarrays (TMAs)

Our normal tissue TMA was composed of eight samples from eight different donors for each of 76 different normal tissue types (608 samples on one slide). The cancer TMAs contained a total of 18,582 primary tumors from 154 tumor types and subtypes. Detailed histopathological and molecular data were available for cancers of the urinary bladder (*n* = 829), kidney (*n* = 1757), colorectum (*n* = 2351), liver (*n* = 301), ovaries (524), pancreas (*n* = 598), thyroid gland (*n* = 518), and the stomach (*n* = 327). Data on pT, pN, grade, and HPV status were available from 902 squamous cell carcinomas of 11 different sites of origin. Clinical follow-up data were accessible from 850 renal cell cancer patients with a median follow-up time of 48 (range 1–250) months. The composition of both normal and cancer TMAs is described in detail in the results section. All samples were from the archives of the Institutes of Pathology, University Hospital of Hamburg, Germany, the Institute of Pathology, Clinical Center Osnabrueck, Germany, and Department of Pathology, Academic Hospital Fuerth, Germany. Tissues were fixed in 4% buffered formalin and then embedded in paraffin. TMA tissue spot diameter was 0.6 mm. The use of archived remnants of diagnostic tissues for manufacturing of TMAs and their analysis for research purposes as well as patient data analysis has been approved by local laws (HmbKHG, §12) and by the local ethics committee (Ethics commission Hamburg, WF-049/09). All work has been carried out in compliance with the Helsinki Declaration.

### Immunohistochemistry

Freshly cut TMA sections were immunostained on one day and in one experiment. Slides were deparaffinized with xylol, rehydrated through a graded alcohol series, and exposed to heat-induced antigen retrieval for 5 min in an autoclave at 121 °C in pH 7.8 DakoTarget Retrieval Solution™ (Agilent, CA, USA; #S2367). Endogenous peroxidase activity was blocked with a Dako Peroxidase Blocking Solution™ (Agilent, CA, USA; #52023) for 10 min. A primary antibody specific for HMGA2 (recombinant rabbit monoclonal, HMV314, cat. # 6632–314-01, ardoci GmbH, Hamburg, Germany) was applied at 37 °C for 60 min at a dilution of 1:200. For the purpose of antibody validation, the normal tissue TMA was also analyzed by the rabbit polyclonal HMGA2 antibody HMGA2-P3 (Biocheck, Forster City, USA) at a dilution of 1:140 and an otherwise identical protocol. A bound antibody was then visualized using the EnVision Kit™ (Agilent, CA, USA; #K5007) according to the manufacturer’s directions. The sections were counterstained with haemalaun. For tumor tissues, the percentage of positive neoplastic cells was estimated, and the staining intensity was semi-quantitatively recorded (0, 1 +, 2 +, 3 +). For statistical analyses, the staining results were categorized into four groups. Tumors without any staining were considered negative. Tumors with 1 + staining intensity in ≤ 70% of cells and 2 + intensity in ≤ 30% of cells were considered weakly positive. Tumors with 1 + staining intensity in > 70% of cells, 2 + intensity in 31–70%, or 3 + intensity in ≤ 30% were considered moderately positive. Tumors with 2 + intensity in > 70% or 3 + intensity in > 30% of cells were considered strongly positive.

### cBioportal HMGA2 RNAseq data

Two datasets on Kidney Renal Clear Cell Carcinoma (TCGA, PanCancer Atlas, 512 samples) and Kidney Renal Clear Cell Carcinoma (TCGA, PanCancer Atlas, 328 samples) [61–70] were selected for survival analysis according to HMGA2 expression on the cBioportal website (https://www.cbioportal.org) [71–73]. The Kaplan–Meier plots were created directly on the cBioportal website. For this purpose, the selected studies were queried for HMGA2 expression using the genomic profile mRNA expression *z*-scores relative to normal samples (log RNA Seq V2 RSEM). Tumors were divided into groups with low and high HMGA2 expression using a *z*-score threshold of ± 2.0. By selecting the menu items comparison/survival and then survival, the Kaplan–Meier plots were generated.

### Statistics

Statistical calculations were performed with JMP17 software (SAS Institute Inc., NC, USA). Contingency tables and the chi^2^-test were performed to search for associations between HMGA2 immunostaining and tumor phenotype. Survival curves were calculated according to Kaplan–Meier. The log-rank test was applied to detect significant differences between groups.

## Results

### Technical issues

A total of 15,915 (85.6%) of 18,582 tumor samples were interpretable in our TMA analysis. Non-interpretable samples demonstrated lack of unequivocal tumor cells or lack of entire tissue spots. A sufficient number of samples (≥ 4) of each normal tissue type was evaluable.

### HMGA2 in normal tissues

**Fig. 2:**
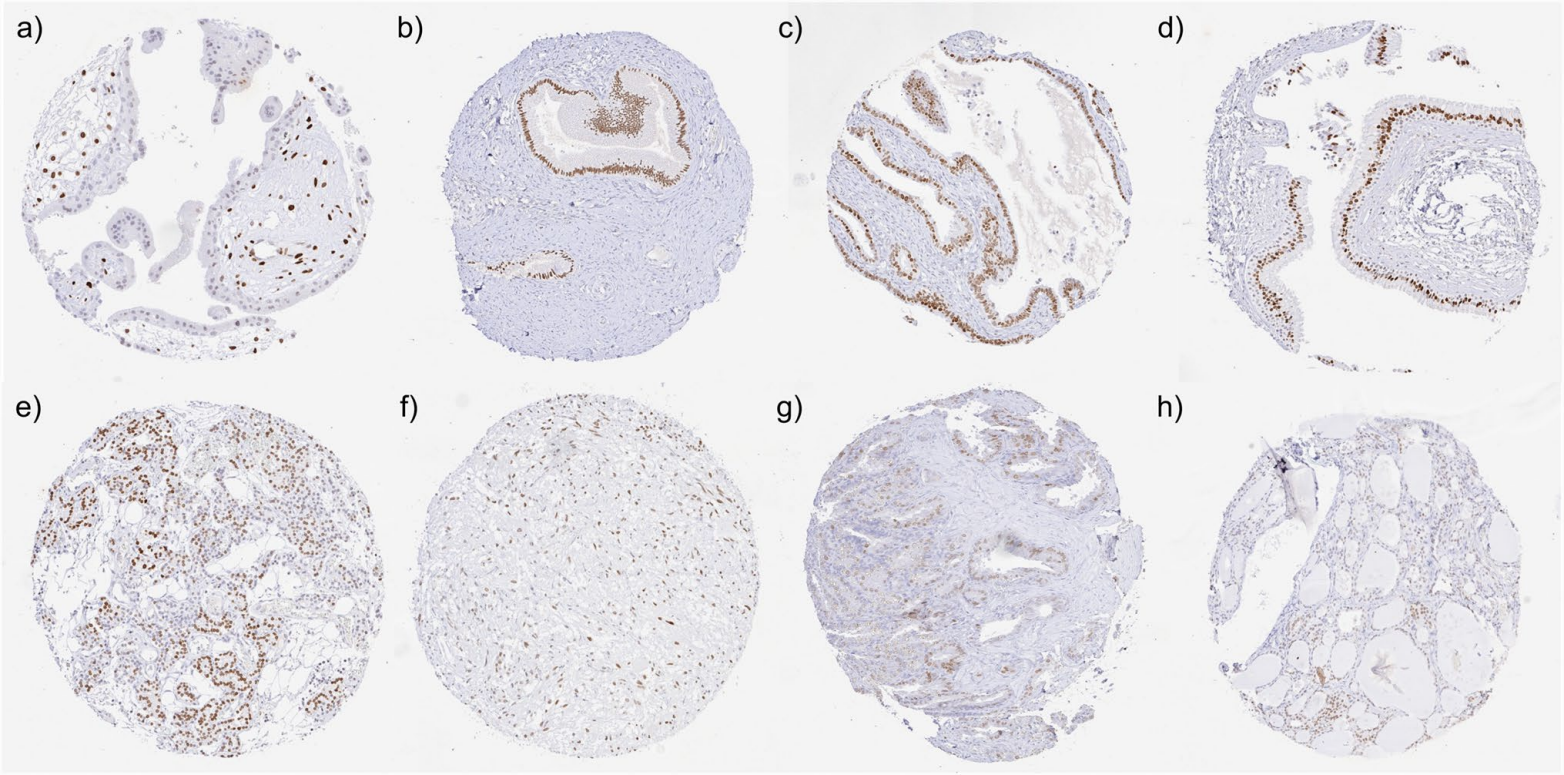
HMGA2 immunostaining of normal tissues. The panels show a distinct nuclear HMGA2 staining of stroma cells of the first trimester placenta **a**, endocervical cells of the uterus **b**, epithelial cells of the fallopian tube **c**, respiratory epithelium of the paranasal sinus **d**, epithelial cells of the parathyroid gland **e**, pituicytes of the neurohypophysis **f**, and a fraction of luminal epithelial cells of the seminal vesicle **g**. HMGA2 staining is only faint in a subset of thyroideal epithelial cells **h**

HMGA2 immunostaining was always nuclear. A strong HMGA2 immunostaining occurred in amnion and stroma cells in the chorionic plate of the placenta, stroma cells of the first trimester placenta, a fraction of luminal epithelial cells of the seminal vesicle, epithelial cells of the endocervix and of the fallopian tube, as well as in most respiratory epithelial cells. A weak or sometimes also moderate HMGA2 staining was also seen in a subset of epithelial cells of the endometrium, a fraction of hemopoietic cells in the bone marrow, trophoblast cells of the mature placenta, a fraction of epithelial cells in the cauda epididymis, spermatocytes and of spermatozoa of the testis, few scattered urothelial cells, some tubular cells of the kidney, a fraction of acinar and ductal cells of the pancreas, intrahepatic bile ducts, a fraction of epithelial cells of the gastrointestinal tract, pituicytes of the neurohypophysis, some epithelial cell groups of the parathyroid gland, follicular cells of the thyroid, and in some myocytes of the skeletal muscle. In some tissues, HMGA2 was particularly seen in areas with tissue damage such as in atrophic renal tubuli or in a pancreatic sample with scar formation and some inflammation. Representative images are shown in Fig. 2. All these cell types were stained by both HMV314 and HMGA2-P3. By using HMGA2-P3, an additional staining was seen in the stratum granulosum of keratinizing squamous epithelium (cytoplasmic) and in epithelial cells of the gastrointestinal tract (apical, granular, cytoplasmic). These stainings were considered antibody specific cross-reactivities of HMGA2-P3. A cytoplasmic staining of some skeletal muscle cells (see panel “x” in Supplementary Fig. 1) was only seen by HMV314 and considered an antibody specific cross-reactivity of HMV314. HMGA2 staining was not seen in salivary and bronchial glands, breast epithelium, heart muscle, smooth muscle, myometrium of the uterus, corpus spongiosum of the penis, ovarian stroma, fat, skin (including hair follicle and sebaceous glands), oral mucosa of the lip, oral cavity, surface epithelium of the tonsil, and transitional mucosa of the anal canal, ectocervix, squamous epithelium of the esophagus, decidua, lymph node, spleen, thymus, tonsil, prostate, lung, adrenal medulla, cerebellum, and the cerebrum. The normal tissue stainings for HMGA2 are summarized in Supplementary Table 2.

### HMGA2 in cancer tissues

**Fig. 3:**
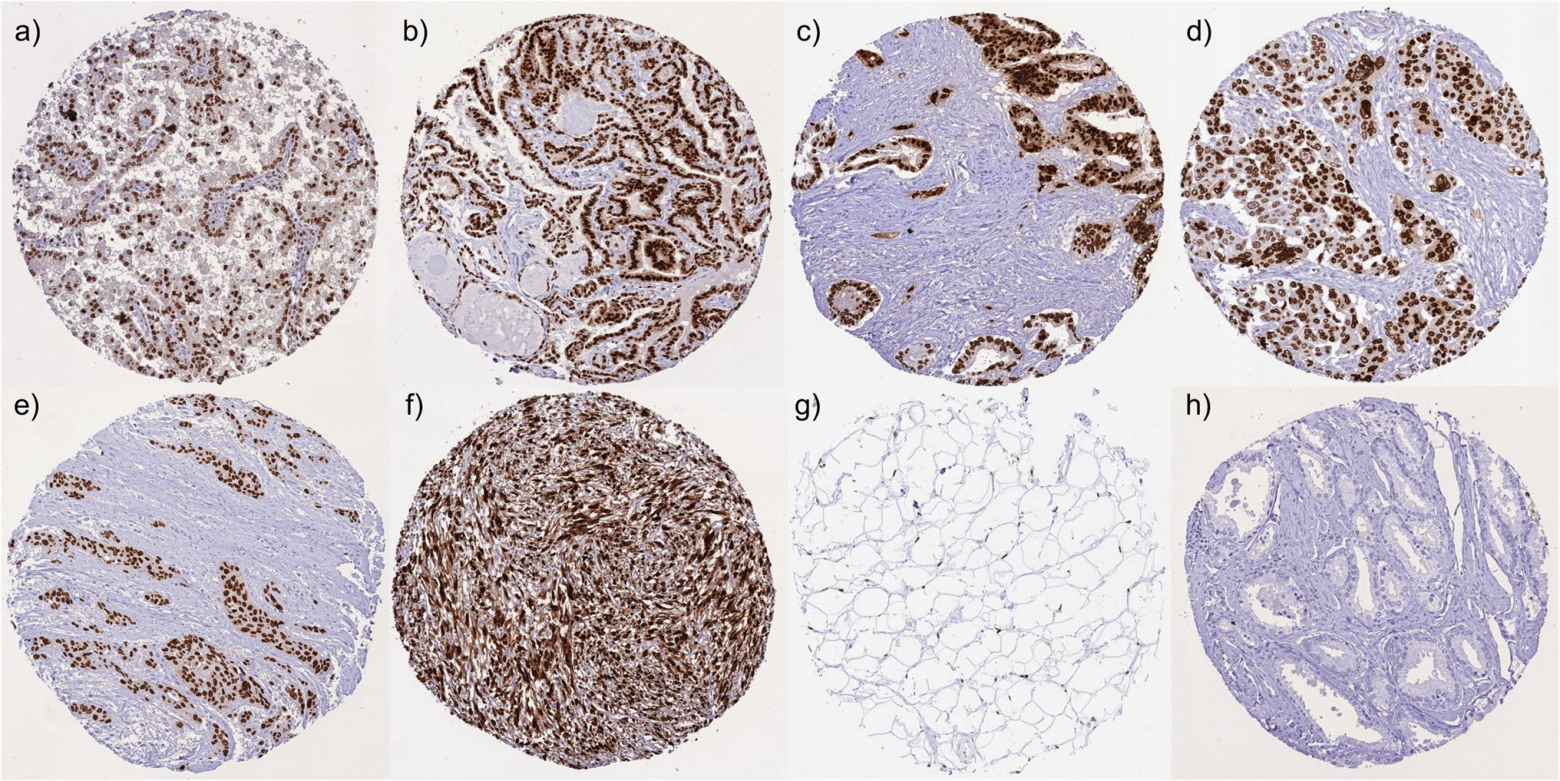
HMGA2 immunostaining of cancers. HMGA2 staining was always nuclear. The panels show a strong HMGA2 positivity in a papillary renal cell carcinoma **a**, a papillary thyroid cancer **b**, a ductal adenocarcinoma of the pancreas **c**, a serous high-grade carcinoma of the uterus **d**, a gastric adenocarcinoma **e**, a liposarcoma **f**, and a lipoma **g**, while HMGA2 staining was absent in a prostatic adenocarcinoma **h**

Nuclear HMGA2 expression was generally markedly higher in cancer than in corresponding normal tissues. HMGA2 staining was found in 5963 (37.5%) of the 15,915 interpretable tumor samples, including 1914 (12%) with weak, 1838 (11.5%) with moderate, and 2211 (13.9%) with strong positivity. A total of 118 of 144 tumor categories showed HMGA2 expression in at least one case, and 92 tumor categories included at least one case with strong HMGA2 staining. The frequency of HMGA2 positivity was particularly high in cancers of the ovary and the endometrium (59–100%), bilio-pancreatic cancers (48.1–73.3%), thyroidal neoplasms (28.4–97.4%), salivary gland neoplasms (15.2–79.2%), non-seminomatous testicular germ cell tumors (3.2–95.6%), colorectal adenomas and adenocarcinomas (65.3–73.2%), gastro-esophageal adenocarcinomas (37.4–75.5%), papillary renal cell carcinomas (61.7%), and in squamous cell carcinomas of various origins (36–81.5%). Tumor entities with a particularly low expression of HMGA2 included prostatic adenocarcinomas (0.4–4%), non-invasive urothelial carcinomas (1.8–4.3%), adenocarcinomas of the breast (1.1–1.5%), and non-Hodgkin’s lymphomas (0%). All data are summarized in Table 1. Representative images of HMGA2 positive tumors are shown in Fig. 3. A graphical representation of a ranking order of HMGA2 positive cancers and of strongly positive cancers is given in Supplementary Fig. 2. The relationship between HMGA2 staining and histopathological or molecular features in individual cancer types is shown in Table 2. High levels of HMGA2 staining were associated with invasive tumor growth of urothelial carcinomas of the urinary bladder (*p* < 0.0001), high grade (*p* < 0.0001), advanced pT (*p* < 0.0001), nodal (*p* = 0.0171), and distant metastasis (*p* < 0.0001) as well as poor overall survival in univariate (*p* < 0.0001; Supplementary Fig. 3) and multivariate analysis (*p* = 0.0374, adjusted for pT and pN) in clear cell RCC, nodal metastasis in papillary RCC (*p* = 0.0099) as well as nodal metastasis in papillary thyroid cancer (*p* = 0.0063). Low HMGA2 expression was also linked to microsatellite instability (*p* = 0.0002) and BRAF mutations (*p* = 0.0018) as well as absence of RAS mutations (*p* < 0.0001) in colorectal cancer and HPV infection in squamous cell carcinomas (*p* < 0.0001, Supplementary Table 1). HMGA2 staining data were unrelated to parameters of cancer aggressiveness in hepatocellular carcinoma, squamous cell carcinomas of different sites of origin, muscle-invasive urothelial carcinoma, serous high-grade carcinoma of the ovary, as well as in gastric and pancreatic adenocarcinoma.

**Table 1:** HMGA2 immunostaining in human tumors

			HMGA2 immunostaining				
	Tumor entity	On TMA (*n*)	Analyzable (*n*)	Negative (%)	Weak (%)	Moderate (%)	Strong (%)
Tumors of the skin	Basal cell carcinoma of the skin	89	72	40.3	34.7	19.4	5.6
	Benign nevus	29	22	50.0	22.7	27.3	0.0
	Squamous cell carcinoma of the skin	145	115	49.6	15.7	18.3	16.5
	Malignant melanoma	65	54	42.6	16.7	18.5	22.2
	Malignant melanoma lymph node metastasis	86	78	37.2	16.7	17.9	28.2
	Merkel cell carcinoma	2	1	100.0	0.0	0.0	0.0
Tumors of the head and neck	Squamous cell carcinoma of the larynx	109	87	32.2	18.4	17.2	32.2
	Squamous cell carcinoma of the pharynx	60	58	41.4	17.2	15.5	25.9
	Oral squamous cell carcinoma (floor of the mouth)	130	117	22.2	18.8	28.2	30.8
	Pleomorphic adenoma of the parotid gland	50	35	42.9	0.0	2.9	54.3
	Warthin tumor of the parotid gland	104	68	86.8	10.3	2.9	0.0
	Acinic cell carcinoma of the salivary gland	181	50	100.0	0.0	0.0	0.0
	Adenocarcinoma NOS of the salivary gland	109	46	84.8	4.3	8.7	2.2
	Adenoid cystic carcinoma of the salivary gland	180	59	42.4	11.9	10.2	35.6
	Basal cell adenocarcinoma of the salivary gland	25	3	66.7	33.3	0.0	0.0
	Basal cell adenoma of the salivary gland	101	24	20.8	16.7	25.0	37.5
	Epithelial-myoepithelial carcinoma of the salivary gland	53	18	55.6	11.1	22.2	11.1
	Mucoepidermoid carcinoma of the salivary gland	343	160	35.0	20.6	26.3	18.1
	Polymorphous adenocarcinoma, low grade, of the salivary gland	41	2	100.0	0.0	0.0	0.0
	Pleomorphic adenoma of the salivary gland	53	26	61.5	0.0	3.8	34.6
Tumors of the lung, pleura and thymus	Adenocarcinoma of the lung	196	173	65.9	9.8	8.1	16.2
	Squamous cell carcinoma of the lung	80	65	18.5	18.5	30.8	32.3
	Mesothelioma, epithelioid	40	33	97.0	0.0	0.0	3.0
	Mesothelioma, biphasic	29	26	53.8	23.1	7.7	15.4
	Thymoma	29	24	37.5	33.3	20.8	8.3
	Lung, neuroendocrine tumor (NET)	29	27	96.3	3.7	0.0	0.0
Tumors of the female genital tract	Squamous cell carcinoma of the vagina	78	57	47.4	12.3	17.5	22.8
	Squamous cell carcinoma of the vulva	157	134	37.3	18.7	20.1	23.9
	Squamous cell carcinoma of the cervix	136	122	62.3	23.0	9.0	5.7
	Adenocarcinoma of the cervix	23	23	65.2	8.7	21.7	4.3
	Endometrioid endometrial carcinoma	338	312	41.0	28.2	19.6	11.2
	Endometrial serous carcinoma	86	71	31.0	16.9	23.9	28.2
	Carcinosarcoma of the uterus	57	42	31.0	9.5	9.5	50.0
	Endometrial carcinoma, high grade, G3	13	13	15.4	38.5	30.8	15.4
	Endometrial clear cell carcinoma	9	8	75.0	12.5	12.5	0.0
	Endometrioid carcinoma of the ovary	130	110	36.4	19.1	23.6	20.9
	Serous carcinoma of the ovary, high grade	580	536	20.9	22.2	24.3	32.6
	Mucinous carcinoma of the ovary	101	84	33.3	13.1	38.1	15.5
	Clear cell carcinoma of the ovary	51	43	86.0	7.0	4.7	2.3
	Carcinosarcoma of the ovary	47	44	13.6	6.8	13.6	65.9
	Granulosa cell tumor of the ovary	44	41	24.4	9.8	36.6	29.3
	Leydig cell tumor of the ovary	4	4	100.0	0.0	0.0	0.0
	Sertoli cell tumor of the ovary	1	1	0.0	0.0	100.0	0.0
	Sertoli Leydig cell tumor of the ovary	3	3	0.0	33.3	66.7	0.0
	Steroid cell tumor of the ovary	3	3	100.0	0.0	0.0	0.0
	Brenner tumor	41	41	80.5	12.2	7.3	0.0
Tumors of the breast	Invasive breast carcinoma of no special type	1764	1685	98.9	0.8	0.2	0.1
	Lobular carcinoma of the breast	363	338	98.5	0.6	0.3	0.6
	Medullary carcinoma of the breast	34	33	100.0	0.0	0.0	0.0
	Tubular carcinoma of the breast	29	23	100.0	0.0	0.0	0.0
	Mucinous carcinoma of the breast	65	54	100.0	0.0	0.0	0.0
	Phyllodes tumor of the breast	50	43	90.7	4.7	2.3	2.3
Tumors of the digestive system	Adenomatous polyp, low-grade dysplasia	50	41	26.8	26.8	41.5	4.9
	Adenomatous polyp, high-grade dysplasia	50	47	21.3	53.2	25.5	0.0
	Adenocarcinoma of the colon	2483	2332	34.7	25.3	25.1	14.8
	Gastric adenocarcinoma, diffuse type	215	174	62.6	9.8	20.7	6.9
	Gastric adenocarcinoma, intestinal type	215	176	39.8	20.5	22.2	17.6
	Gastric adenocarcinoma, mixed type	62	59	45.8	18.6	20.3	15.3
	Adenocarcinoma of the esophagus	83	53	24.5	24.5	34.0	17.0
	Squamous cell carcinoma of the esophagus	76	46	37.0	30.4	6.5	26.1
	Squamous cell carcinoma of the anal canal	91	81	59.3	16.0	7.4	17.3
	Cholangiocarcinoma	58	52	51.9	5.8	17.3	25.0
	Gallbladder adenocarcinoma	51	48	33.3	18.8	10.4	37.5
	Gallbladder Klatskin tumor	42	42	42.9	21.4	11.9	23.8
	Hepatocellular carcinoma	312	312	73.7	5.8	5.4	15.1
	Ductal adenocarcinoma of the pancreas	659	554	26.7	20.2	20.8	32.3
	Pancreatic/ampullary adenocarcinoma	98	88	42.0	27.3	15.9	14.8
	Acinar cell carcinoma of the pancreas	18	17	76.5	11.8	5.9	5.9
	Gastrointestinal stromal tumor (GIST)	62	53	52.8	5.7	9.4	32.1
	Appendix, neuroendocrine tumor (NET)	25	17	94.1	5.9	0.0	0.0
	Colorectal, neuroendocrine tumor (NET)	12	11	90.9	9.1	0.0	0.0
	Ileum, neuroendocrine tumor (NET)	53	49	98.0	0.0	2.0	0.0
	Pancreas, neuroendocrine tumor (NET)	101	96	81.3	4.2	5.2	9.4
	Colorectal, neuroendocrine carcinoma (NEC)	14	13	61.5	7.7	7.7	23.1
	Ileum, neuroendocrine carcinoma (NEC)	8	5	80.0	0.0	20.0	0.0
	Gallbladder, neuroendocrine carcinoma (NEC)	4	4	50.0	25.0	0.0	25.0
	Pancreas, neuroendocrine carcinoma (NEC)	14	13	61.5	23.1	7.7	7.7
Tumors of the urinary system	Non-invasive papillary urothelial carcinoma, pTa G2 low grade	87	71	95.8	0.0	4.2	0.0
	Non-invasive papillary urothelial carcinoma, pTa G2 high grade	80	69	95.7	4.3	0.0	0.0
	Non-invasive papillary urothelial carcinoma, pTa G3	126	109	98.2	1.8	0.0	0.0
	Urothelial carcinoma, pT2-4 G3	735	480	71.7	5.0	6.0	17.3
	Squamous cell carcinoma of the bladder	22	21	23.8	19.0	14.3	42.9
	Small cell neuroendocrine carcinoma of the bladder	5	5	100.0	0.0	0.0	0.0
	Sarcomatoid urothelial carcinoma	25	21	9.5	19.0	23.8	47.6
	Urothelial carcinoma of the kidney pelvis	62	58	56.9	6.9	10.3	25.9
	Clear cell renal cell carcinoma	1287	1195	92.8	2.6	1.8	2.8
	Papillary renal cell carcinoma	368	332	38.3	29.5	20.5	11.7
	Clear cell (tubulo) papillary renal cell carcinoma	26	22	81.8	9.1	0.0	9.1
	Chromophobe renal cell carcinoma	170	154	92.2	4.5	1.9	1.3
	Oncocytoma of the kidney	257	230	97.4	2.2	0.4	0.0
Tumors of the male genital organs	Adenocarcinoma of the prostate, Gleason 3 + 3	83	74	100.0	0.0	0.0	0.0
	Adenocarcinoma of the prostate, Gleason 4 + 4	80	66	98.5	1.5	0.0	0.0
	Adenocarcinoma of the prostate, Gleason 5 + 5	85	75	96.0	4.0	0.0	0.0
	Adenocarcinoma of the prostate (recurrence)	258	236	99.6	0.4	0.0	0.0
	Small cell neuroendocrine carcinoma of the prostate	2	1	100.0	0.0	0.0	0.0
	Seminoma	682	663	81.3	10.4	3.6	4.7
	Embryonal carcinoma of the testis	54	49	26.5	40.8	24.5	8.2
	Leydig cell tumor of the testis	31	31	96.8	0.0	3.2	0.0
	Sertoli cell tumor of the testis	2	2	100.0	0.0	0.0	0.0
	Spermatocytic tumor of the testis	1	1	100.0	0.0	0.0	0.0
	Yolk sac tumor	53	45	4.4	13.3	26.7	55.6
	Teratoma	53	43	81.4	0.0	0.0	18.6
	Squamous cell carcinoma of the penis	92	86	64.0	12.8	7.0	16.3
Tumors of endocrine organs	Adenoma of the thyroid gland	113	90	38.9	12.2	13.3	35.6
	Papillary thyroid carcinoma	391	332	9.0	2.4	6.3	82.2
	Follicular thyroid carcinoma	154	111	38.7	7.2	13.5	40.5
	Medullary thyroid carcinoma	111	81	71.6	3.7	7.4	17.3
	Parathyroid gland adenoma	43	37	70.3	5.4	18.9	5.4
	Anaplastic thyroid carcinoma	45	39	2.6	5.1	10.3	82.1
	Adrenal cortical adenoma	48	47	100.0	0.0	0.0	0.0
	Adrenal cortical carcinoma	27	27	37.0	33.3	25.9	3.7
	Pheochromocytoma	51	50	94.0	6.0	0.0	0.0
Tumors of hematopoetic and lymphoid tissues	Hodgkin ‘s lymphoma	103	81	77.8	11.1	11.1	0.0
	Small lymphocytic lymphoma, B-cell type (B-SLL/B-CLL)	50	50	100.0	0.0	0.0	0.0
	Diffuse large B cell lymphoma (DLBCL)	113	113	100.0	0.0	0.0	0.0
	Follicular lymphoma	88	88	100.0	0.0	0.0	0.0
	T-cell non-Hodgkin's lymphoma	25	25	100.0	0.0	0.0	0.0
	Mantle cell lymphoma	18	18	100.0	0.0	0.0	0.0
	Marginal zone lymphoma	16	16	100.0	0.0	0.0	0.0
	Diffuse large B-cell lymphoma (DLBCL) in the testis	16	16	100.0	0.0	0.0	0.0
	Burkitt lymphoma	5	5	100.0	0.0	0.0	0.0
Tumors of soft tissue and bone	Granular cell tumor	23	21	100.0	0.0	0.0	0.0
	Leiomyoma	50	48	75.0	8.3	6.3	10.4
	Leiomyosarcoma	94	82	74.4	6.1	4.9	14.6
	Lipoma	12	12	25.0	0.0	25.0	50.0
	Liposarcoma	96	84	45.2	7.1	2.4	45.2
	Malignant peripheral nerve sheath tumor (MPNST)	15	12	16.7	16.7	16.7	50.0
	Myofibrosarcoma	26	26	46.2	15.4	15.4	23.1
	Angiosarcoma	42	37	62.2	5.4	13.5	18.9
	Angiomyolipoma	91	87	98.9	1.1	0.0	0.0
	Dermatofibrosarcoma protuberans	21	15	100.0	0.0	0.0	0.0
	Ganglioneuroma	14	13	100.0	0.0	0.0	0.0
	Kaposi sarcoma	8	5	80.0	0.0	20.0	0.0
	Neurofibroma	117	114	86.0	7.0	5.3	1.8
	Sarcoma, not otherwise specified (NOS)	74	67	55.2	11.9	4.5	28.4
	Paraganglioma	41	41	95.1	4.9	0.0	0.0
	Ewing sarcoma	23	18	72.2	5.6	16.7	5.6
	Rhabdomyosarcoma	7	7	57.1	0.0	14.3	28.6
	Schwannoma	122	113	61.9	18.6	15.0	4.4
	Synovial sarcoma	12	10	40.0	20.0	30.0	10.0
	Osteosarcoma	19	15	60.0	6.7	13.3	20.0
	Chondrosarcoma	15	8	25.0	0.0	25.0	50.0
	Rhabdoid tumor	5	4	25.0	0.0	0.0	75.0
	Solitary fibrous tumor	17	17	100.0	0.0	0.0	0.0

**Table 2:** HMGA2 immunostaining and cancer phenotype

				HMGA2 IHC result				
			*n*	Negative (%)	Weak (%)	Moderate (%)	Strong (%)	*P*
Urinary bladder cancer	Tumor stage	pTa G2 low	270	94.1	2.6	0.7	2.6	*p* < 0.0001
		pTa G2 high	81	88.9	4.9	3.7	2.5	
		pTa G3	11	81.8	9.1	0.0	9.1	
		pT2	213	56.8	11.3	5.2	26.8	
		pT3	263	57.4	9.5	5.7	27.4	
		pT4	128	64.1	8.6	6.3	21.1	
	Nodal stage	pN0	282	56.4	12.1	5.7	25.9	0.0746
		pN +	199	67.8	8.0	3.5	20.6	
Clear cell renal cell cancers	ISUP stage	1	268	98.5	1.1	0.4	0.0	< 0.0001
		2	398	97.7	1.3	0.8	0.3	
		3	264	88.6	4.2	3.8	3.4	
		4	74	64.9	5.4	6.8	23.0	
	Fuhrman grade	1	65	98.5	1.5	0.0	0.0	< 0.0001
		2	679	97.5	1.6	0.7	0.1	
		3	294	89.8	4.1	2.7	3.4	
		4	89	65.2	5.6	7.9	21.3	
	Thoenes grade	1	352	98.3	1.1	0.6	0.0	< 0.0001
		2	490	93.7	3.5	1.8	1.0	
		3	96	74.0	4.2	6.3	15.6	
	UICC stage	1	323	96.3	2.2	0.9	0.6	< 0.0001
		2	38	94.7	5.3	0.0	0.0	
		3	94	90.4	6.4	1.1	2.1	
		4	71	76.1	4.2	7.0	12.7	
	Tumor stage	pT1	671	96.1	2.1	1.2	0.6	< 0.0001
		pT2	134	94.8	3.0	0.7	1.5	
		pT3-4	327	85.6	3.4	3.7	7.3	
	Nodal stage	pN0	174	93.1	2.9	1.7	2.3	0.0171
		pN +	25	72.0	4.0	8.0	16.0	
	Dist. mets stage	pM0	111	97.3	2.7	0.0	0.0	< 0.0001
		pM +	91	76.9	3.3	5.5	14.3	
Papillary renal cell cancers	ISUP stage	1	36	41.7	22.2	22.2	13.9	0.1809
		2	132	39.4	31.8	22.7	6.1	
		3	80	43.8	27.5	17.5	11.3	
		4	7	42.9	14.3	0.0	42.9	
	Fuhrman grade	1	2	50.0	50.0	0.0	0.0	0.0605
		2	178	37.1	30.9	23.6	8.4	
		3	82	47.6	28.0	14.6	9.8	
		4	11	36.4	9.1	9.1	45.5	
	Thoenes grade	1	55	38.2	29.1	16.4	16.4	0.102
		2	156	42.9	30.1	20.5	6.4	
		3	16	31.3	12.5	31.3	25.0	
	UICC stage	1	100	35.0	31.0	24.0	10.0	0.7069
		2	13	46.2	15.4	15.4	23.1	
		3	5	60.0	20.0	20.0	0.0	
		4	11	45.5	18.2	18.2	18.2	
	Tumor stage	pT1	201	38.8	31.3	21.9	8.0	0.1879
		pT2	48	41.7	20.8	20.8	16.7	
		pT3-4	33	39.4	27.3	12.1	21.2	
	Nodal stage	pN0	25	28.0	32.0	36.0	4.0	0.0099
		pN +	14	28.6	21.4	7.1	42.9	
	Dist. mets stage	pM0	26	23.1	42.3	19.2	15.4	0.2085
		pM +	11	36.4	9.1	27.3	27.3	
Papillary thyroid cancer	Tumor stage	pT1	138	12.3	2.9	5.1	79.7	0.6388
		pT2	73	11.0	2.7	9.6	76.7	
		pT3-4	92	14.1	1.1	3.3	81.5	
	Nodal stage	pN0	84	11.9	3.6	8.3	76.2	0.0063
		pN +	112	3.6	0.0	4.5	92.0	
Colorectal carcinoma	Tumor stage	pT1	86	36.0	27.9	24.4	11.6	0.9875
		pT2	435	36.8	25.1	23.4	14.7	
		pT3	1256	35.4	26.1	24.2	14.3	
		pT4	433	34.2	24.7	26.1	15.0	
	Nodal stage	pN0	1160	34.8	26.2	25.4	13.5	0.5108
		pN +	1046	35.8	25.0	23.9	15.4	
	Tumor localization	Left colon	1224	35.0	25.1	25.3	14.6	0.1489
		Right colon	459	39.9	25.9	21.1	13.1	
	MMR status	Defective	86	51.2	30.2	12.8	5.8	0.0002
		Proficient	1147	33.6	25.1	25.7	15.6	
	RAS mutation status	Mutated	358	29.1	25.1	31.6	14.2	< 0.0001
		Wildtype	469	40.3	25.4	19.0	15.4	
	BRAF mutation status	Mutated	22	50.0	31.8	0.0	18.2	0.0018
		Wild-type	125	36.0	22.4	31.2	10.4	
Hepatocellular carcinoma	Tumor stage	pT1	79	77.2	3.8	1.3	17.7	0.0372
		pT2	88	68.2	6.8	5.7	19.3	
		pT3-4	64	60.9	12.5	12.5	14.1	
	Nodal stage	pN0	78	70.5	9	7.7	12.8	0.1317
		pN +	43	51.2	14	7	27.9	
	Grade	G 1	38	84.2	0	2.6	13.2	0.0738
		G 2	136	66.2	9.6	8.1	16.2	
		G 3	54	68.5	5.6	3.7	22.2	
Serous ovarian cancer	Tumor stage	pT1	33	18.2	27.3	33.3	21.2	0.3809
		pT2	43	23.3	14.0	23.3	39.5	
		pT3	266	19.9	24.8	21.4	33.8	
	Nodal stage	pN0	81	14.8	23.5	25.9	35.8	0.3943
		pN1	164	23.2	24.4	20.1	32.3	
Pancreatic adenocarcinoma	Tumor stage	pT1	9	22.2	0	44.4	33.3	0.1614
		pT2	62	30.6	14.5	21	33.9	
		pT3	347	26.8	23.1	20.7	29.4	
		pT4	26	26.9	19.2	7.7	46.2	
	Grade	G1	13	30.8	23.1	30.8	15.4	0.6958
		G2	313	25.6	21.7	19.2	33.5	
		G3	95	31.6	18.9	20	29.5	
	Nodal stage	pN0	86	30.2	24.4	14	31.4	0.3394
		pN +	356	26.4	20.5	22.2	30.9	
Stomach cancer	Laurén type	Diffuse	91	68.1	9.9	15.4	6.6	
		Intestinal	89	42.7	23.6	19.1	14.6	
		Mixed	59	45.8	18.6	20.3	15.3	
	Tumor stage	pT1-2	61	67.2	14.8	13.1	4.9	0.1027
		pT3	123	44.7	18.7	21.1	15.4	
		pT4	125	50.4	20.0	16.8	12.8	
	Nodal stage	pN0	82	56.1	14.6	19.5	9.8	0.4405
		pN +	226	49.1	20.4	16.8	13.7	
	Mismatch repair status	MMR defective	40	57.5	17.5	20.0	5.0	0.2527
		MMR proficient	260	46.9	19.2	18.8	15.0	
Squamous cell carcinomas	Tumor stage	pT1	253	44.3	19.4	15.4	20.9	0.2686
		pT2	262	44.7	16.4	19.5	19.5	
		pT3	131	34.4	22.1	16.8	26.7	
		pT4	127	36.2	16.5	18.9	28.3	
	Nodal stage	pN0	296	37.8	22.0	17.9	22.3	0.274
		pN +	299	40.1	15.7	19.1	25.1	
	Grade	G1	32	53.1	18.8	15.6	12.5	0.2747
		G2	362	40.6	17.1	19.3	22.9	
		G3	244	44.7	19.3	12.7	23.4	

### cBioportal survival analysis of HMGA2 expression

The Kaplan plots of clear cell and papillary renal cell carcinomas are shown in Supplementary Fig. 4. High levels of HMGA2 RNA expression occurred in a small subset (43 of 480) clear cell carcinomas and were closely associated with poor survival (*p* < 0.0001, Supplementary Fig. 4a). The prognostic impact of high HMGA2 expression was markedly lower in 282 papillary carcinomas (*p* = 0.0176).

## Discussion

Our data provide a comprehensive overview on HMGA2 expression in normal and cancerous tissues and show that HMGA2 is often overexpressed in tumors. Considering the controversial data from the literature, such information could hardly be compiled from the literature (Fig. 1). That HMGA2 positivity was seen in 118 of 144 tumor entities including 92 tumor entities with at least one strongly positive case is suggestive for an important role of HMGA2 in many cancer entities. Most normal cell types that often develop HMGA2 positive cancers were largely HMGA2 negative at the selected experimental conditions. This pinpoints towards a critical role of HMGA2 overexpression in neoplastic transformation of many human cell types. The biological importance of HMGA2 is further supported by significant associations between HMGA2 overexpression and features of tumor progression in several tumor types.

The prognostic role of HMGA2 upregulation was particularly strong in clear cell renal cell carcinoma (ccRCC). Although there were only 4.6% of cancers with moderate or strong HMGA2 positivity, these tumors almost exclusively showed unfavorable tumor parameters and poor outcomes. A strong and independent prognostic role of HMGA2 overexpression was also found in a recent IHC study on 162 patients with ccRCC [74]. Data from the “The Cancer Genome Atlas” TCGA database also show a striking prognostic role of high HMGA2 expression [71–73]. In line with our data, the TCGA dataset identified only a small set of HMGA2 positive cancers with a markedly adverse prognosis in ccRCC (Supplementary Fig. 2a) while the group of positive cancers was much larger in papillary RCC, where the prognostic impact was less stringent (Supplementary Fig. 2b). The significant increase of HMGA2 positivity from non-invasive (5.9–18.2% positive) to invasive urothelial carcinomas (35.9–43.2%) is another example where HMGA2 upregulation is linked to cancer progression. Earlier studies had also reported associations between HMGA2 overexpression and aggressive cancer features in breast cancer [37], esophageal [29], gastric [27], pancreatic [14, 25, 30], pulmonary [31], and colorectal adenocarcinoma [34], squamous cell cancer of the tongue [39] and of the esophagus [35], papillary renal cell carcinoma (RCC) [36], hepatocellular carcinoma [38], retinoblastoma [32, 33], glioma [28, 40], and osteosarcoma [26].

The absence of a relationship between the HMGA2 expression levels and parameters of cancer aggressiveness in hepatocellular carcinomas, squamous cell carcinomas of different sites of origin, muscle-invasive urothelial carcinomas, serous high-grade carcinomas of the ovary, as well as in colorectal, gastric, and pancreatic adenocarcinomas in our study demonstrates, however, that HMGA2 upregulation does not play a clinically important prognostic role in all cancer types. The role of HMGA2 critically depends on specific cellular environments fits well with the strong inverse correlation with MSI in colorectal cancer (CRC) and HPV infection in squamous cell carcinomas. Both observations agree with earlier data. Wang et al. described an inverse relationship between MSI and elevated HMGA2 in CRC [75], and Günther et al. found an inverse relationship between HPV infection and elevated HMGA2 in squamous cell carcinoma of the head and neck [76]. Of note, the presence of high levels of HMGA2 RNA and protein in some tumor types has prompted the search for potential inhibitors and anti-HMGA2 therapies. Strategies for targeting HMGA2 include small molecules such as netropsin, metformin or the antifungal agent ciclopirox (CPX) [77–80], and small interfering RNA (siRNA) [81].

Our data suggest considerable potential utility of HMGA2 IHC in surgical pathology, mainly as a marker for neoplastic transformation in various cell types/organs. HMGA2 positivity by IHC may, for example, support the diagnosis of neoplastic lesions in the thyroid and in mesenchymal tissues. In our study, 91% of papillary thyroid carcinomas, 61.3% of follicular carcinomas, and 61.1% of follicular adenomas were HMGA2 positive, but none of eight normal thyroid samples. These positivity rates are somewhat higher than the 60–72.2% of papillary carcinomas, 19.1–35.3% of follicular carcinomas, and 0–41.7% of follicular adenomas found by others [48, 82, 83]. HMGA2 IHC may be especially useful in thyroidal cytology, where the sensitivity is only 40–95% for recognizing papillary carcinomas [84, 85] and 42–83% in follicular carcinomas [85, 86]. A strong HMGA2 overexpression, even in benign mesenchymal neoplasms, can enable a clear-cut distinction of neoplastic from non-neoplastic tissues. This may, for example, facilitate the identification of leiomyoma fragments in endometrial biopsies or the distinction of lipoma from normal fat tissue. In agreement with our data, HMGA2 expression had previously been described in 9.5–96.2% of leiomyomas [52, 87] and in 54% of 13 bronchial lipomas [45]. That HMGA2 positivity was not seen in 80 renal angiomyolipomas suggests that the lipomatous component of these tumors develops differently than in conventional lipomas. To safely exploit the promising diagnostic potential of HMGA2 in surgical pathology, it will be necessary to expand the analysis of non-neoplastic conditions and to define reproducible thresholds to define HMGA2 positivity for diagnostic purposes.

The high prevalence of HMGA2 expression in many tumor types argues against a strong role of HMGA2 IHC for the distinction of tumor entities. Some exceptions may exist, however. For example, the HMGA2 positivity rate was markedly higher in papillary carcinomas of the kidney (61.7%) than in clear cell (7.2%) and in chromophobe cancers (7.8%), and in yolk sac tumors (95.6%) or embryonal carcinomas (73.5% positive) than in seminomas (18.7% positive) of the testis. Because 63% of 27 adrenocortical carcinomas were HMGA2 positive while all 47 adrenocortical adenomas were HMGA2 negative in our study, HMGA2 IHC may possibly serve as a marker of malignancy in adrenocortical neoplasms. The diagnostic value of HMGA2 IHC in these applications awaits further investigation.

Considering the large scale of our study, our assay was extensively validated by comparing our IHC findings in normal tissues with data obtained by another independent anti-HMGA2 antibody and RNA data derived from three different publicly accessible databases [88–91]. This procedure is recommended by the international working group of antibody validation [92]. To ensure as broad a possible range of proteins to be tested for a possible cross-reactivity, 76 different normal tissue categories were included in this analysis. The validity of our IHC assay was supported by the detection of HMGA2 positivity in almost all tissues with documented HMGA2 RNA expression (thyroid, pituitary gland, stomach, colorectum, appendix, small intestine, duodenum, pancreas, testis, cervix uteri, endometrium, fallopian tube, and bone marrow). True expression of HMGA2 in all cell types found to be HMGA2 positive is corroborated by identical staining of every positive cell type by the independent polyclonal antibody HMGA2-P3. Confirmed HMGA2 positive cell types also included several tissues without previously documented HMGA2 RNA expression. It appears likely that HMGA2 RNAs remained undetected in previous analyses because the positive cell types (bile ducts in the liver, some tubules and collecting ducts in the kidney, distinct groups of epithelial cells in the parathyroid, amnion and stroma cells of the placenta, acinar glandular cells of the seminal vesicle, and a fraction of epithelial cells in the cauda epididymis) made up for a too small subset of the respective organs. The ovary and the skin were the only organs with previously described HMGA2 RNA expression but absence of HMGA2 staining by both antibodies. Additional staining of the granular cell layer in keratinizing squamous epithelium and of a granular cytoplasmic apical staining in GIT epithelium, which were only seen by HMGA2-P3, were considered antibody-specific cross-reactivities of HMGA2-P3. Cytoplasmic staining of some skeletal muscle cells was considered a cross-reactivity of HMV314.

The use of tissue microarrays (TMAs) was essential for conducting this study on more than 15,000 tumors. A key advantage of the TMA approach is its high level of standardization which even includes equal quantities of tissue analyzed per cancer. Tissue quantity is important because cancers are potentially heterogeneous and patchy immunostaining often occurs. The likelihood of finding focal staining increases with the quantity of tumor cells analyzed [93–96]. In that respect, the variable tumor quantity on conventional whole sections ranging from less than 1 mm^2^ to more than 6 cm^2^ represents a significant potential bias for study result [97]. This is all more true since even a very large whole section of 3 cm × 2 cm × 2.5 µm only represents an 1/44,000 of a cancer of 5 cm in diameter. The diameter of 0.6 mm per tumor selected for this study may represent the smallest size of tissue needed in a small biopsy for categorizing a cancer. It is to be expected that larger tumor samples may contain focal HMGA2 staining in a fraction of cases. The significance of such findings remains to be clarified in further studies.

In summary, our data identify HMGA2 as a protein that is frequently overexpressed in tumors of all kinds and highlight several potential diagnostic applications for HMGA2 IHC, especially as a marker for neoplastic transformation. Drug candidates that directly or indirectly target HMGA2 protein may be worth considering in HMGA2-overexpressing cancers.

## Supplementary Information

Below is the link to the electronic supplementary material.

Supplementary file1 (PDF 950 KB)

Supplementary file2 (PDF 42 KB)

Supplementary file3 (PDF 18 KB)

Supplementary file4 (PDF 176 KB)

Supplementary file5 (DOCX 14 KB)

Supplementary file6 (DOCX 14.1 KB)
