# Supplementary figures and images for: High mobility group protein 2 (HMGA2) is highly expressed in a broad range of benign and malignant tumors
Source: Virchows Arch. 2025 Jun 16;487(1):183–201. doi: 10.1007/s00428-025-04142-1 (PMC12289817; doi:10.1007/s00428-025-04142-1)

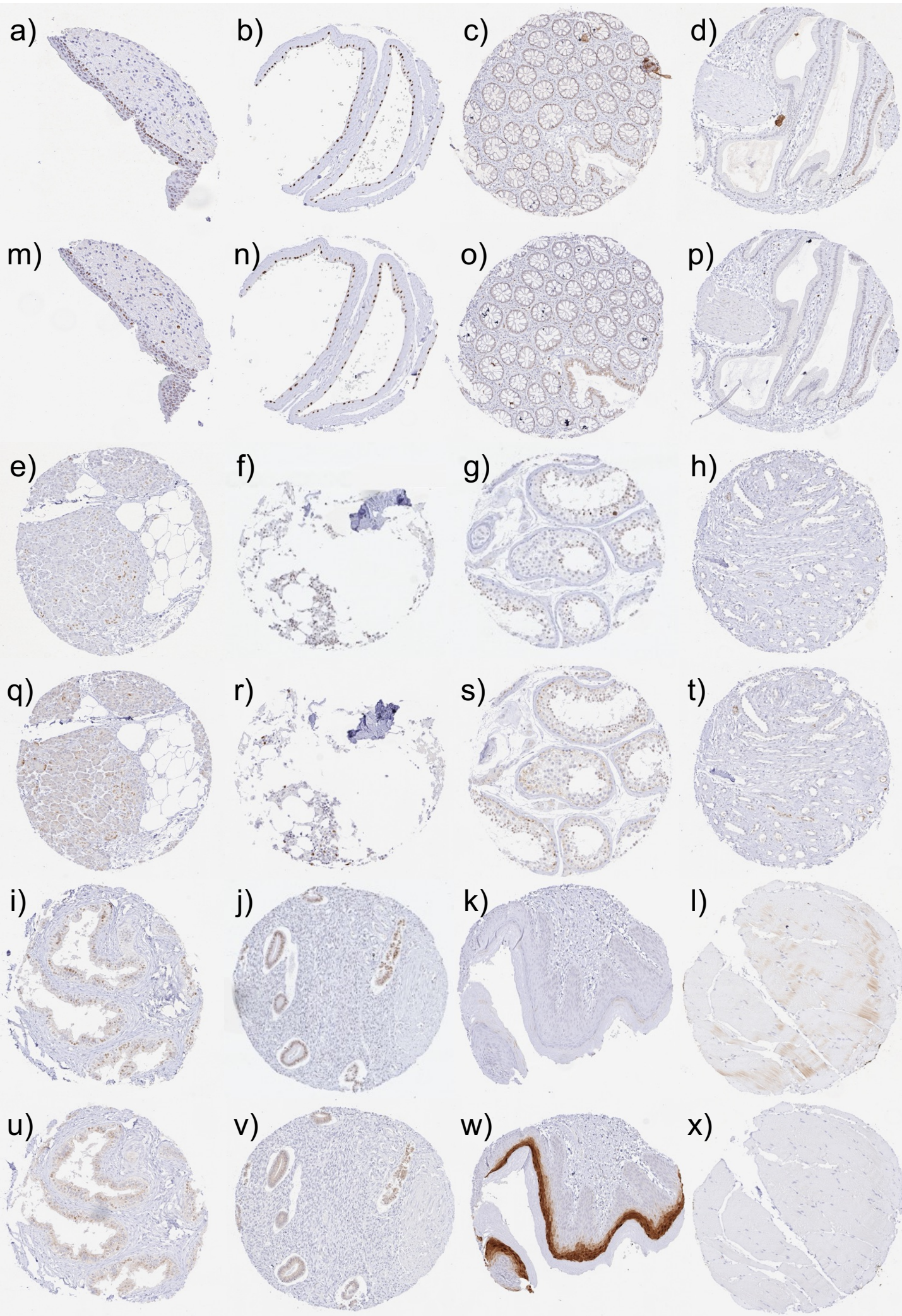

Supplement: Supplementary file 1 — Supplementary file1 (PDF 950 KB) [file 428_2025_4142_MOESM1_ESM.pdf]

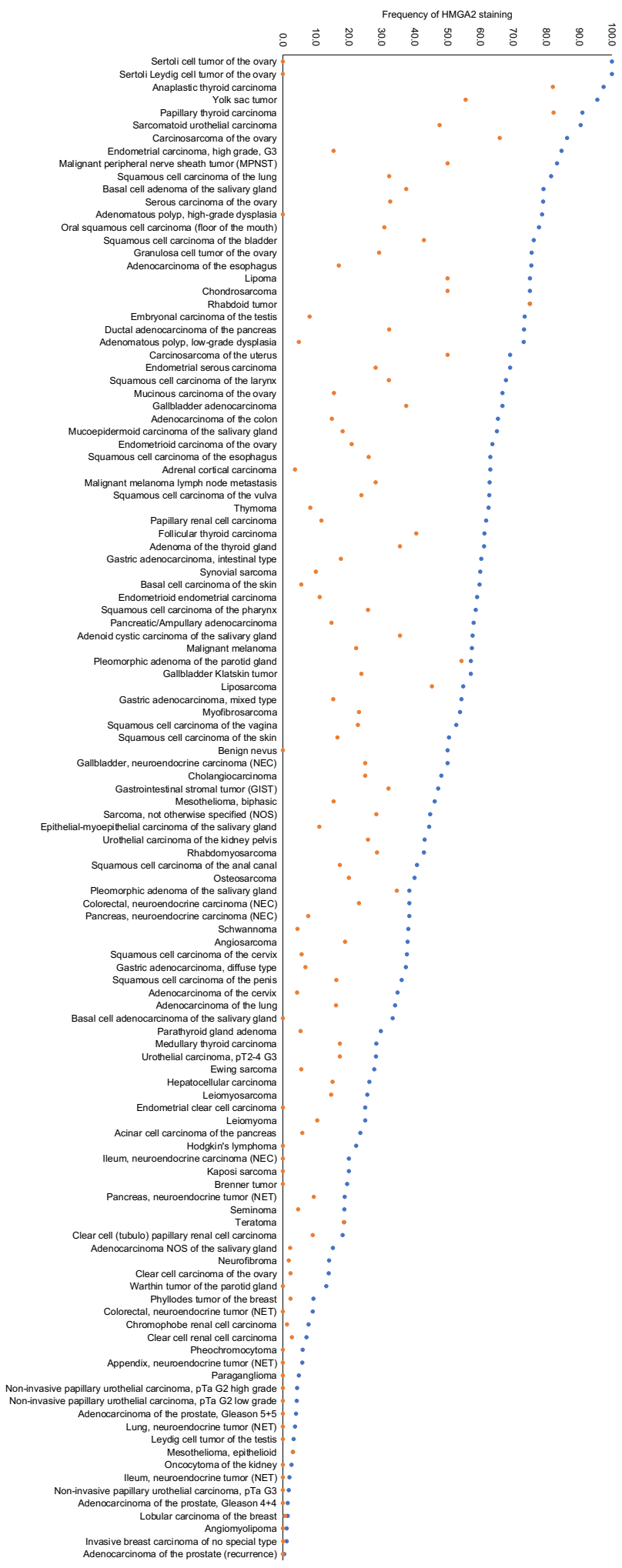

Supplement: Supplementary file 2 — Supplementary file2 (PDF 42 KB) [file 428_2025_4142_MOESM2_ESM.pdf]

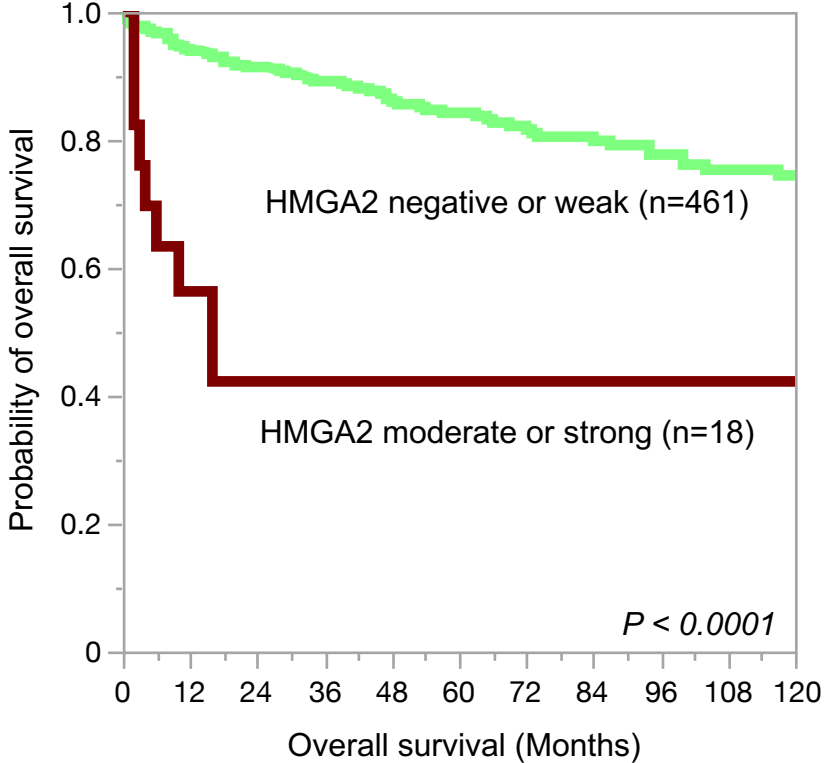

Supplement: Supplementary file 3 — Supplementary file3 (PDF 18 KB) [file 428_2025_4142_MOESM3_ESM.pdf]

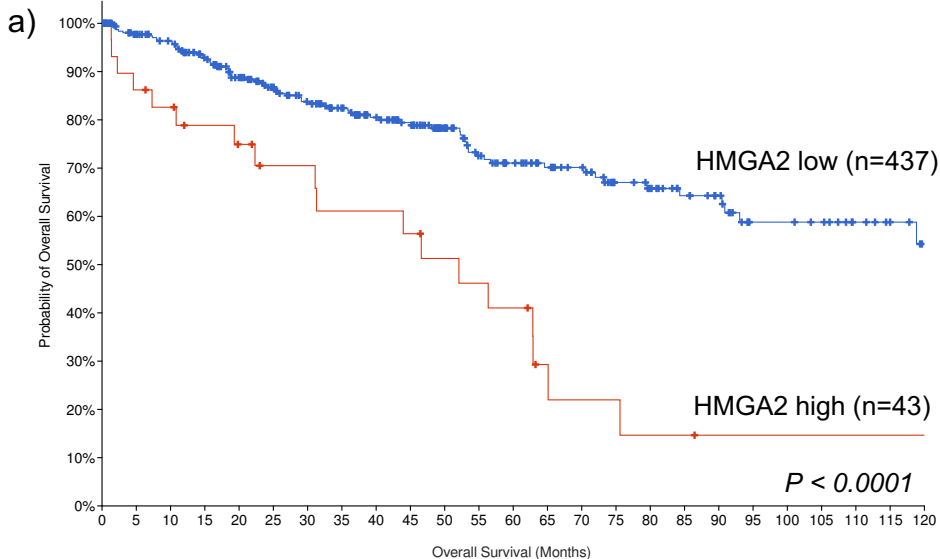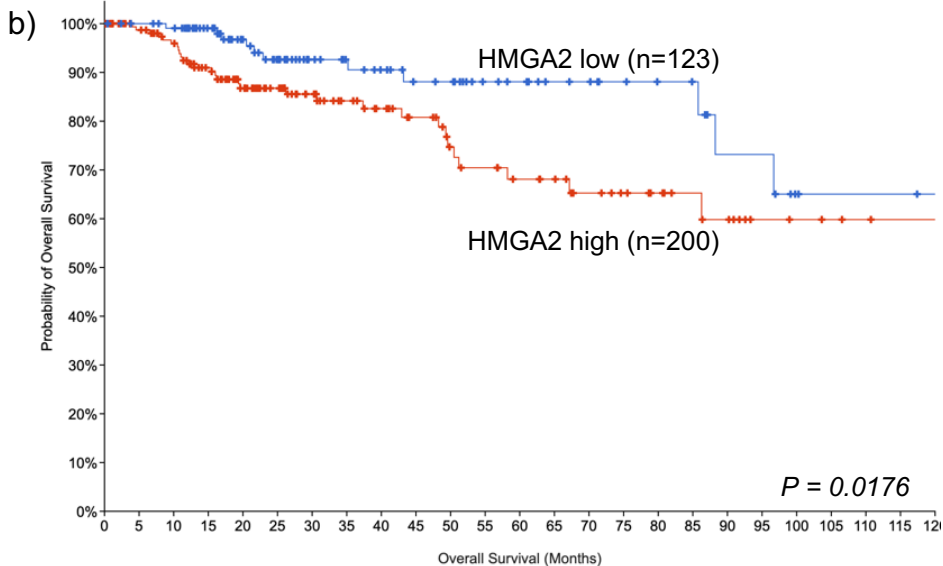

Supplement: Supplementary file 4 — Supplementary file4 (PDF 176 KB) [file 428_2025_4142_MOESM4_ESM.pdf]
