# Supplementary material for: High mobility group protein 2 (HMGA2) is highly expressed in a broad range of benign and malignant tumors
Source: Virchows Arch. 2025 Jun 16;487(1):183–201. doi: 10.1007/s00428-025-04142-1 (PMC12289817; doi:10.1007/s00428-025-04142-1)
Supplement: Supplementary file 5 — Supplementary file5 (DOCX 14 KB) [file 428_2025_4142_MOESM5_ESM.docx]

| **Supplementary Table 2:** HMGA2 immunostaing in human normal tissues | |  |
| --- | --- | --- |
|  |  |  |
| **Tissue Type** | **Cell type** | **HMGA2 Immunostaining** |
| **Placenta** | Amnion and stromal cells | strong |
|  | Stromal cells of the first trimester | strong |
|  | Trophoblasts of the mature placenta | weak to moderate |
| **Female/Male genital tract** | Luminal epithelial cells of the seminal vesicle | strong |
|  | Epithelial cells of the endocervix | strong |
|  | Epithelial cells of the fallopian tube | strong |
|  | Epithelial cells in the cauda epididymis* | weak to moderate |
|  | Spermatocytes and of spermatozoa of the testis | weak to moderate |
| **Respiratory tract** | Respiratory epithelial cells | strong |
| **Urinary system** | Urothelial cells | weak to moderate |
|  | Tubular cells of the Kidney* | weak to moderate |
|  | Atrophic renal tubuli (damaged areas) | weak to moderate |
| **Digestive system** | Intrahepatic bile ducts | weak to moderate |
|  | Epithelial cells of the gastrointestinal tract* | weak to moderate |
|  | Acinar and ductal cells of the pancreas* | weak to moderate |
|  | Pancreatic sample with scar formation and inflammation | weak to moderate |
| **Endocrine system** | Pituicytes of the neurohypophysis | weak to moderate |
|  | Epithelial cell groups of the parathyroid gland* | weak to moderate |
|  | Follicular cells of the thyroid | weak to moderate |
| **Hematopioetic System** | Hemopoietic cells in the bone marrow* | weak to moderate |
| **Muscular system** | Myocytes of the skeletal muscle | weak to moderate |
| *fraction/subset of cells |  |  |
