# Supplementary material for: High mobility group protein 2 (HMGA2) is highly expressed in a broad range of benign and malignant tumors
Source: Virchows Arch. 2025 Jun 16;487(1):183–201. doi: 10.1007/s00428-025-04142-1 (PMC12289817; doi:10.1007/s00428-025-04142-1)
Supplement: Supplementary file 6 — Supplementary file6 (DOCX 14.1 KB) [file 428_2025_4142_MOESM6_ESM.docx]

|  | **HPV status** | **n** | **HMGA2 IHC (%)** | | | | **p** |
| --- | --- | --- | --- | --- | --- | --- | --- |
|  |  |  | **negative** | **weak** | **moderate** | **strong** |  |
|  |  |  |  |  |  |  |  |
| All squamous cell cancers | negativ | 314 | 35.0 | 15.6 | 22.3 | 27.1 | <0.0001 |
|  | positive | 239 | 59.8 | 18.8 | 10.5 | 10.9 |  |
|  |  |  |  |  |  |  |  |
| Oral squamous cell carcinoma | negativ | 64 | 14.1 | 14.1 | 35.9 | 35.9 | 0.0187 |
|  | positive | 12 | 58.3 | 8.3 | 16.7 | 16.7 |  |
|  |  |  |  |  |  |  |  |
| Squamous cell carcinoma of the pharynx | negativ | 20 | 15.0 | 20.0 | 20.0 | 45.0 | 0.0138 |
|  | positive | 35 | 57.1 | 11.4 | 14.3 | 17.1 |  |
|  |  |  |  |  |  |  |  |
| Squamous cell carcinoma of the larynx | negativ | 49 | 34.7 | 16.3 | 20.4 | 28.6 | 0.9048 |
|  | positive | 8 | 37.5 | 25.0 | 12.5 | 25.0 |  |
|  |  |  |  |  |  |  |  |
| Squamous cell carcinoma of the cervix | negativ | 10 | 60.0 | 0.0 | 30.0 | 10.0 | 0.0607 |
|  | positive | 68 | 60.3 | 25.0 | 8.8 | 5.9 |  |
|  |  |  |  |  |  |  |  |
| Squamous cell carcinoma of the vagina | negativ | 15 | 46.7 | 0.0 | 26.7 | 26.7 | 0.0536 |
|  | positive | 14 | 42.9 | 28.6 | 21.4 | 7.1 |  |
|  |  |  |  |  |  |  |  |
| Squamous cell carcinoma of the vulva | negativ | 53 | 37.7 | 17.0 | 18.9 | 26.4 | 0.4784 |
|  | positive | 25 | 44.0 | 28.0 | 12.0 | 16.0 |  |
|  |  |  |  |  |  |  |  |
| Squamous cell carcinoma of the penis | negativ | 29 | 62.1 | 10.3 | 3.4 | 24.1 | 0.1949 |
|  | positive | 45 | 73.3 | 13.3 | 6.7 | 6.7 |  |
|  |  |  |  |  |  |  |  |
| Squamous cell carcinoma of the skin | negativ | 37 | 35.1 | 16.2 | 24.3 | 24.3 | 0.4323 |
|  | positive | 1 | 0.0 | 0.0 | 100.0 | 0.0 |  |
|  |  |  |  |  |  |  |  |
| Squamous cell carcinoma of the anal canal | negativ | 5 | 60.0 | 0.0 | 20.0 | 20.0 | 0.4091 |
|  | positive | 31 | 71.0 | 12.9 | 3.2 | 12.9 |  |
|  |  |  |  |  |  |  |  |
